# Response to letter “transgenerational adverse effects of valproate can’t be by-passed”

**DOI:** 10.1017/S109285292500029X

**Published:** 2025-06-02

**Authors:** Roger S. McIntyre

**Affiliations:** 1 Department of Psychiatry, University of Toronto, Toronto, ON, Canada; 2 Department of Pharmacology, University of Toronto, Toronto, ON Canada

**Keywords:** Valproate, teratogenicity, bipolar, epilepsy, neural tube defect

Thank you to Martin et al. for bringing to our attention their report on the transgenerational adverse effects of valproate.^1^ Their report, a survey of 108 individuals from 90 families who experienced “complications” from valproate exposure in utero, is of scientific interest and is critically important in our contemplations of valproate and risk/benefit decisions as part of shared decision-making in considering this agent. Their report, data gathered when individuals were parents themselves as part of the organization Aide aux Parents d’Enfants Souffrants du Syndrome de l’AntiConvulsivant (APESAC), a charity created to provide personal assistance and support to families suffering complications due to valproate exposure during pregnancy, concluded that 53% of their children (n = 187) had major congenital malformations and/or neurodevelopmental disorders (e.g. cognitive, language problems). The aforementioned report suggests genetic/epigenetic modifications due to valproate may be transgenerational.

The article by Martin et al. was not included in our report wherein we summarized extant literature (n = 122 studies) and endeavored to provide a quantitative and updated estimation of valproate-associated anatomical, behavioral, and cognitive teratogenicity.^2^ We did not include the report of Martin et al. as our eligibility for study inclusion was explicitly to look at offspring effects and we did not cover transgenerational outcomes. We observed that prenatal valproate exposure was highly associated with dose-dependent anatomical, behavioral as well as cognitive teratogenicity (odds ratio [OR; 2.47–9.30; 1.70–4.38 and 2.4–4.48, respectively). We also identified increased risk for valproate-associated teratogenicity when prescribed with other antiepileptic drugs and could not identify compelling replicated evidence that folic acid supplementation attenuated or abrogated the risk of valproate-associated teratogenicity. Although we did not include the paper by Martin et al., their results comport with our findings of the very serious safety concerns as it relates to teratogenicity.

The report from Martin et al. is alarming from the point of view of transgenerational risk as it relates to valproate exposure. If replicated, it would further strengthen calls to significantly reduce valproate exposure in persons of reproductive age.^3^ ^,^^4^ Multiple non-competing hypotheses are proposed to mechanistically explain the association between valproate exposure and teratogenicity including, but not limited, to histone deacetylation inhibition (HDACi), folic acid deficiency, and increase in oxidative stress.^5^ For example, HDACi would be predicted to affect DNA methylation and alter apoptotic intracellular machinery affecting neuronal growth and development. The results from Martin et al. suggest that the genetic/epigenetic effects of valproate may not be confined to exposed populations only and may be transmitted transgenerationally. Extant literature as it pertains to valproate-associated teratogenicity is compelling, fit for purpose, and should affect practitioner behavior. We agree with recommendations that valproate should only be considered for persons of childbearing potential if other options have been carefully considered and are not viable alternatives.^3^
